# Feasibility and Acceptability of Music Imagery and Listening Interventions for Analgesia: Protocol for a Randomized Controlled Trial

**DOI:** 10.2196/38788

**Published:** 2022-09-22

**Authors:** Kristin M Story, Dawn M Bravata, Sheri L Robb, Sally Wasmuth, James E Slaven, Leah Whitmire, Barry Barker, Tetla Menen, Matthew J Bair

**Affiliations:** 1 Center for Health Information and Communication (CHIC) Health Services Research & Development (HSRD) Richard L Roudebush Veterans Affairs Medical Center Indianapolis, IN United States; 2 Expanding Expertise through E-health Network Development (EXTEND) Quality Enhancement Research Initiative (QUERI) Veterans Affairs Health Services Research and Development (HSR&D) Indianapolis, IN United States; 3 Departments of Medicine and Neurology Indiana University School of Medicine Indianapolis, IN United States; 4 Regenstrief Institute Inc Indianapolis, IN United States; 5 Indiana University School of Nursing Indianapolis, IN United States; 6 Department of Occupational Therapy Indiana University School of Health and Human Sciences Indianapolis, IN United States; 7 Department of Biostatistics and Health Data Science Indiana University School of Medicine Indianapolis, IN United States; 8 Creative Forces National Endowment for the Arts Henry M Jackson Foundation for the Advancement of Military Medicine Inc Indianapolis, IN United States; 9 Department of Medicine Indiana University School of Medicine Indianapolis, IN United States

**Keywords:** chronic pain, music therapy, veterans, clinical trial, music imagery, pilot study, feasibility, acceptability, mobile phone

## Abstract

**Background:**

Chronic pain and access to care are identified as critical needs of the Veterans Health Administration. Music imagery and music listening interventions have shown promise as effective nonpharmacological options for pain management. However, most studies have focused on acute pain, passive music experiences, and in-person delivery.

**Objective:**

In this study, we aimed to examine the feasibility and acceptability of 2 music interventions delivered through telehealth for chronic musculoskeletal pain, trial design, and theoretical model before conducting a fully powered efficacy or comparative effectiveness trial.

**Methods:**

FAMILIA (Feasibility and Acceptability of Music Imagery and Listening Interventions for Analgesia) is a 3-arm, parallel group, pilot trial. A total of 60 veterans will be randomized to one of the three conditions: music imagery, music listening, or usual care. Aim 1 is to test the feasibility and acceptability of a multicomponent, interactive music imagery intervention (8-weekly, individual sessions) and a single-component, minimally interactive music learning intervention (independent music listening). Feasibility metrics related to recruitment, retention, engagement, and completion of the treatment protocol and questionnaires will be assessed. Up to 20 qualitative interviews will be conducted to assess veteran experiences with both interventions, including perceived benefits, acceptability, barriers, and facilitators. Interview transcripts will be coded and analyzed for emergent themes. Aim 2 is to explore the effects of music imagery and music listening versus usual care on pain and associated patient-centered outcomes. These outcomes and potential mediators will be explored through changes from baseline to follow-up assessments at 1, 3, and 4 months. Descriptive statistics will be used to describe outcomes; this pilot study is not powered to detect differences in outcomes.

**Results:**

Recruitment for FAMILIA began in March 2022, and as of July 2022, 16 participants have been enrolled. We anticipate that enrollment will be completed by May 2023. We expect that music imagery and music listening will prove acceptable to veterans and that feasibility benchmarks will be reached. We hypothesize that music imagery and music listening will be more effective than usual care on pain and related outcomes.

**Conclusions:**

FAMILIA addresses four limitations in music intervention research for chronic pain: limited studies in veterans, evaluation of a multicomponent music intervention, methodological rigor, and internet-based delivery. Findings from FAMILIA will inform a fully powered trial to identify putative mechanisms and test efficacy.

**Trial Registration:**

ClinicalTrials.gov NCT05426941; https://tinyurl.com/3jdhx28u

**International Registered Report Identifier (IRRID):**

DERR1-10.2196/38788

## Introduction

### Chronic Pain

Chronic pain, a persistent problem for United States veterans, has a reported prevalence as high as 65.5% in the veteran population and is associated with limitations in mobility and daily activities, dependence on opioids, anxiety and depression, and poor perceived health [1,2]. Chronic pain is often inadequately treated with analgesics alone and results in substantial disability, reduced health-related quality of life, and increased health care use and costs. Musculoskeletal pain accounts for more than half of all patients with pain disorders presenting for clinical care and analgesic treatment is insufficient for many patients [3,4]. To improve pain-related outcomes, the Department of Veterans Affairs (VA) recommends an integrative approach to pain management, including nonpharmacological interventions [5,6].

### Music Therapy and Pain

Music therapy interventions target biopsychosocial outcomes and show promise as nonpharmacological options for pain. Music therapists use active (music making) and receptive (music listening) interventions to support pain management; however, much of the music and chronic illness literature comprises studies that use receptive interventions [7,8]. Studies of music listening interventions have demonstrated statistically significant reductions in self-reported pain, emotional distress, and opioid use, but the findings are inconsistent and although there are a few studies that address patients with chronic pain, the focus has been primarily on acute pain [9-13]. A majority of these studies evaluated recorded music listening programs, with inconsistent findings likely because of an absence of theoretical frameworks to guide music selection and tailored delivery, and educational components to encourage independent use of music. Music listening interventions often involve minimal interaction with a music therapist, and are primarily designed to be patient self-directed, empowering patients to use music whenever needed. However, some patients with complex pain and psychological comorbidities may need a more intensive and interactive music therapy intervention that includes ongoing support, symptom monitoring, and additional treatment components, such as imagery and verbal processing, to potentially enhance the therapeutic effects of music listening.

Compared with recorded listening programs, therapist-led music therapy interventions involve more interactive approaches to music listening that provide education about the therapeutic potential of music and often integrate additional treatment components (eg, lyric discussion, drawing or journaling) to address additional biopsychosocial factors that contribute to chronic pain [14,15]. Music imagery, a receptive music therapy intervention that combines music listening, imagery, and verbal processing, has been used to address a variety of patient needs (eg, symptoms related to posttraumatic stress disorder [PTSD], cancer, mood disorders, and chronic pain [16-19]). In pilot studies with nonpain populations (eg, PTSD, health care providers), participants found that music imagery enhanced their coping skills and ability to self-regulate [16,17]. Both music listening and music imagery have shown benefits for patients with fibromyalgia and other chronic pain illnesses, including improved well-being and decreased pain, anxiety, and depression [20-22]. Taken as a whole, the music therapy literature suggests that music listening and music imagery may improve pain outcomes, but gaps in the literature include inadequate evaluations among patients with chronic pain, theoretically grounded interventions, and lack of methodological rigor.

### Telehealth Delivery

Although VA recommends using nonpharmacological interventions for chronic pain, there are significant barriers that limit access to creative arts interventions, including music therapy (eg, rural and homebound veterans [23]). Virtual delivery of health care can improve access, especially for the older adults, the mobility-impaired, and those living in rural areas or those with transportation barriers [24,25]. Case studies of virtually delivered music therapy have described veterans’ endorsement of telehealth sessions and patient-reported benefits [26]. Although these studies provide compelling preliminary data, more research is needed to develop and test specific music interventions for chronic pain that can be delivered through telehealth. In this context, we will evaluate 2 music interventions (music listening and music imagery) for veterans with chronic musculoskeletal pain.

### Study Innovation

The FAMILIA (Feasibility and Acceptability of Music Imagery and Listening Interventions for Analgesia) study addresses four limitations in music intervention research for chronic pain: few studies in veterans, evaluation of multicomponent music interventions, methodological rigor, and virtual delivery. Most previous studies described a passive, one-session, single-component music listening experience for patients with acute pain [9-13]. In contrast, the FAMILIA study will evaluate 2 music interventions in veterans. Compared with music listening, less is known about multicomponent music interventions, which we hypothesize are more robust in reducing pain owing to greater treatment intensity and more frequent interactions with a music therapist. The FAMILIA study will assess outcomes (feasibility, acceptability, pain, and associated outcomes) of a single-component, minimally interactive intervention (music listening) and a multicomponent, interactive intervention (music imagery).

**Figure 1:**
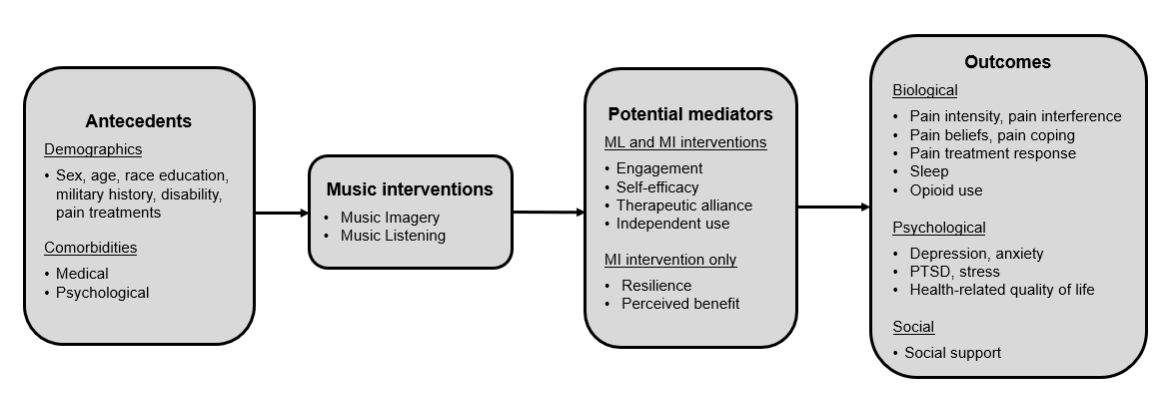
Study conceptual framework. MI: music imagery; ML: music listening; PTSD: posttraumatic stress disorder.

Systematic reviews of music interventions for pain cite a lack of methodological rigor because of poorly described interventions, a lack of theoretical foundation, and no reporting of adverse events [27,28]. The FAMILIA study will address these methodological concerns in several ways. First, we will adhere to reporting guidelines for music-based interventions developed by Robb et al [29]. Second, we will use a theoretically based conceptual framework (Figure 1) to explore the hypothesized mechanism of action and outcomes. Third, we will systematically monitor for specific potential adverse events in both interventions.

FAMILIA addresses important knowledge gaps regarding virtual delivery of music therapy interventions. The COVID-19 pandemic has rapidly accelerated the widespread use of telehealth services for multiple chronic conditions, including chronic pain, but there are few published studies to guide virtual delivery of music therapy. We recently conducted the first enterprise-wide survey examining current practices in telehealth delivery by VA creative arts therapists. Initial results indicated that 76% of creative arts therapists have delivered virtual sessions, with 74% delivering >50 sessions in the past year [30]. Telehealth provides greater access, convenience, continuity of care, and support for veterans [31,32]. Early adopters of telehealth music therapy have provided general instructive examples, but there is a need for rigorous research of music interventions delivered virtually to determine their feasibility, acceptability, and efficacy [23,26,33]. We expect that our study will inform evidence-based practice and help expand access to music interventions for chronic pain.

### Rationale and Specific Aims

The objective of this randomized controlled trial (RCT) is to examine feasibility and acceptability of the interventions, trial design, and theoretical model before conducting a fully powered efficacy or comparative effectiveness trial. Findings from FAMILIA will be used to refine the interventions and theoretical model, allowing for a comprehensive analysis of proposed mediators and outcomes in a subsequent trial. The 2 specific aims and the related key questions are shown in Textbox 1.

**Study aims and key questions.**

**Aims and Questions**

Study aim 1: test the feasibility and acceptability of a multicomponent, interactive music imagery intervention and a single-component, minimally interactive music listening intervention.

- What percentage of veterans consent to study participation, attend intervention sessions, and complete the treatment protocol and scheduled outcome assessments?
- What are veteran experiences with both interventions?
- What aspects of the interventions do veterans perceive to be most or least helpful and most or least liked?
- What are barriers and facilitators to study participation?
- How did the music interventions compare to other chronic pain treatments already tried?

Study aim 2: explore the effects of both music interventions (music imagery and music listening) vs usual care on pain and associated patient-centered outcomes.

- What are the changes from baseline to follow-up assessments (1, 3, and 4 months) on pain and associated patient-centered outcomes?
- What are the changes in proximal and distal mediators, and do they vary based on group assignment?
- What proportion of veterans achieve a clinically meaningful change in pain intensity and pain interference?

## Methods

### Study Design

**Figure 2:**
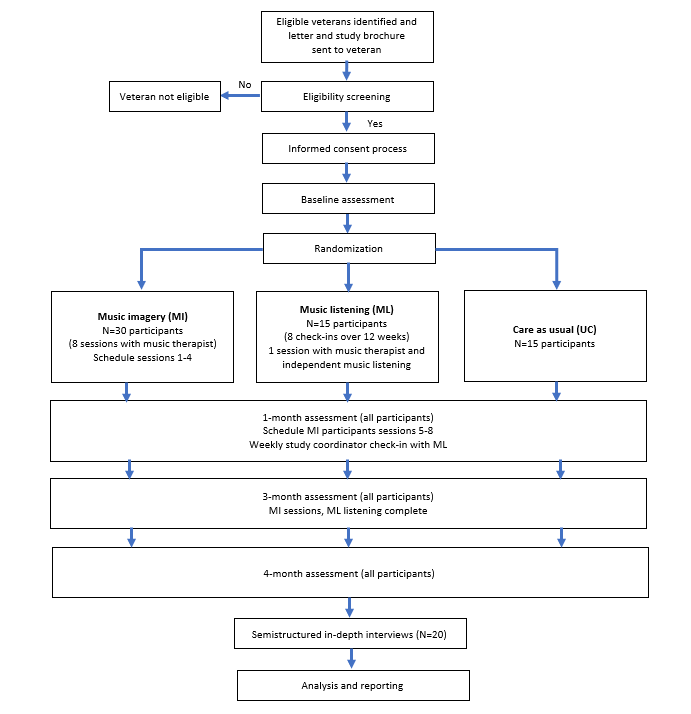
Flowchart of study design.

The FAMILIA study is a 3-arm, parallel group, RCT, as shown in Figure 2. After providing informed consent and completing their baseline assessment, participants will be randomized in a 2:1:1 allocation ratio to 1 of 3 groups. Twice as many participants will be randomized to the music imagery group than other groups because a key goal of this pilot study is to gain experience with and learn about patients’ perceptions of a multicomponent, interactive music therapy intervention. The goal sample size is 60 participants, with approximately 30 participants in the music imagery arm, 15 participants in the music listening arm, and 15 participants in the usual care arm. A sample size of 15 to 20 per group is sufficient to assess the feasibility and acceptability in pilot trials [34].

Randomization will be stratified according to biological sex (male vs female). Within strata, randomization with block sizes of 9 will be executed to ensure a balance of key baseline characteristics (eg, baseline pain intensity).

### Sample Size and Randomization

#### Recruitment

The study population will include veterans receiving care through the Richard L Roudebush VA Medical Center, Indianapolis, Indiana, United States. Inclusion criteria are veterans with chronic musculoskeletal pain of at least moderate severity (graded by ≥5 on a 0-10 scale); access to a PC, tablet, or smartphone; and ability to pass a technology screening assessment. Veterans with serious or unstable medical or psychiatric illness, housing insecurity that would prevent study participation, or suicidal ideation with current intent are excluded from participation. Veterans with hearing or cognitive impairment are also excluded because of possible interference with music listening or abstract thinking needed for music imagery work.

Veterans will be recruited from primary and specialty care clinics. Study enrollment and intervention activities will primarily be delivered virtually through Microsoft Teams, which is a VA-approved audio and video platform. Participants will be provided the options of reviewing informed consent, completing their baseline assessment, and attending their initial music imagery or music listening session in person to build rapport and identify any technology concerns. Participation and refusal rates will be tracked throughout enrollment and reported in aggregate by study group.

#### Participant Recruitment Strategies

Recruitment strategies mirror those that have been successful in multiple previous pain trials [35-38]. Primary care physicians will be informed of study details and asked to refer patients they feel could potentially participate in the study. Potential participants will be identified by querying the VA’s electronic medical record system to create a master list of veterans with the International Classification of Diseases 9th and 10th editions (ICD 9/10) codes for chronic musculoskeletal pain (ie, low back pain, neck pain or cervicalgia, fibromyalgia, and osteoarthritis). An invitation letter will be mailed to qualifying veterans to describe the study. Potential participants will be contacted by phone after the letter is mailed to assess eligibility and determine their interest in participating. Clinical staff may refer veterans who have not received a letter to the research team, and self-referral is possible by patients responding to a study brochure or advertisement displayed in primary care, orthopedic, rheumatology, pain, and rehabilitation clinics. The research team will determine eligibility by applying the inclusion and exclusion criteria to potential participants during an eligibility evaluation.

### Interventions

#### Music Imagery Group

Up to 30 participants will be randomized to receive 8 weekly music imagery sessions over 8 to 12 weeks. Allotted time will be up to 12 weeks for treatment to allow for rescheduling if needed. Each session will last approximately 45 minutes. music imagery is derived from Guided Imagery and Music, 1 of the 5 international models of music therapy practice and the most well-known receptive music therapy model [39]. The intervention uses the participant’s relationship to music to connect and enhance inner resources and provide a music resource for self-care [40]. Inner resources such as creativity, courage, and serenity connect an individual to their resiliency in the face of conflict, or to overcome some adversity. Resilience is the ability to maintain positive emotional functioning despite physical or psychological challenges [41]. An inner resource may be a visual image of a nurturing figure, the feeling of calming music, or colors that represent strength or a sense of peace. The primary aim in music imagery for chronic pain management is to address participants’ relationship to pain, their ability to interact adaptively with chronic pain, and to use supportive and accessible tools (eg, music to self-regulate through relaxation, distraction, and increased connection to inner resources) to manage pain and related psychological symptoms (eg, depression and anxiety) more effectively.

**Figure 3:**
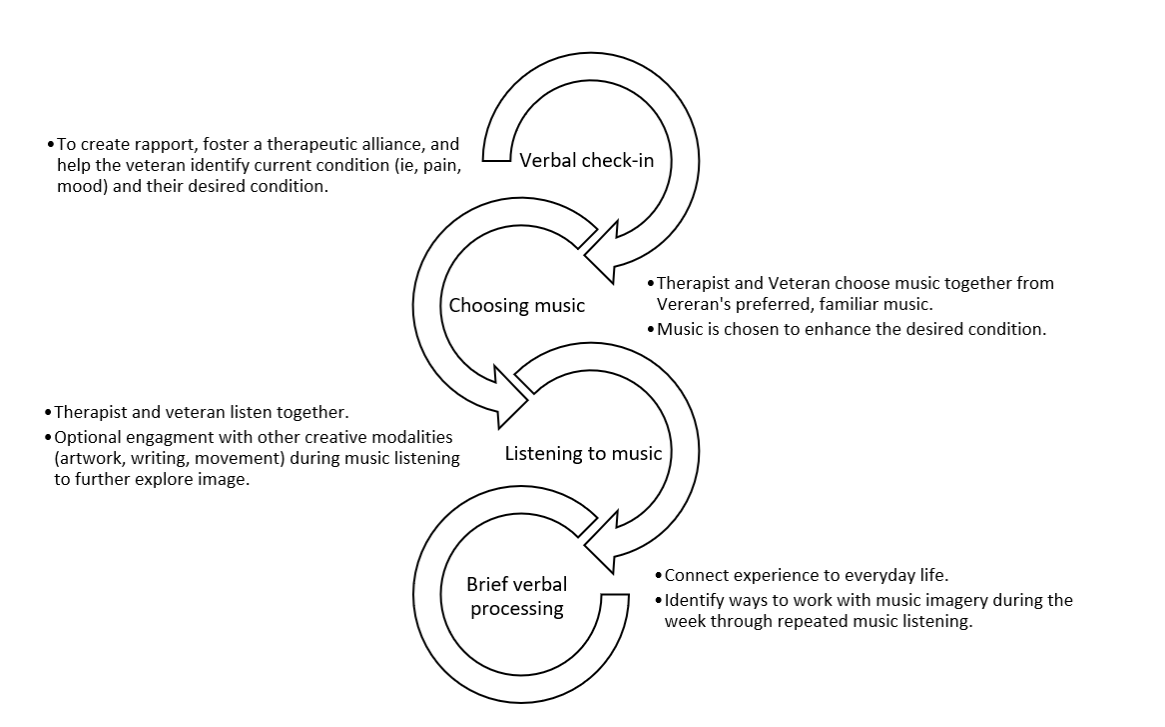
Music imagery intervention steps. MI: music imagery.

Each music imagery session will be delivered by a board-certified music therapist with specialized training in music imagery. Participants will receive individual therapist-directed sessions. The sessions include 4 steps as illustrated in Figure 3. There is a strong educational component to music imagery, partly met through weekly homework, during which participants are encouraged to *play* with the music and imagery used during the therapist-led session. In this context, *play* refers to the continued engagement with the creative process through repeated listening of music coupled with other modalities (eg, drawing, creative writing, and dancing). The music therapist and veteran identify specific ways to engage with music and imagery between sessions.

#### Music Listening Group

In the music listening arm, a music therapist will meet with the veteran once to identify musical tastes, preferences, and activities; musical memories and social influences; and relationships between music, health, and quality of life [42]. From this meeting, an electronic playlist will be compiled for the veteran to listen to during the 12-week treatment period. The veteran will be asked to maintain a music listening log, including how long they listen, what they listen to, and any reactions to the music (eg, benefits and triggers). Music listening time will not be prescribed, but participants will be encouraged to keep track of their listening to music during the week. Our rationale for including the music listening intervention is to explore whether there is a clinically meaningful change in measures and perceived benefits from a less interactive, less intensive music intervention.

A member of the research team will check in with music listening participants (weekly for the first 3 weeks following their initial session, then biweekly following the 1-month assessment [approximately 7 check-ins in total]) to monitor safety, adherence (ie, time listening to music), and identify other issues.

#### Control Group: Usual Care

Participants in the usual care group (as is the case for the music imagery and music listening groups) may receive analgesics and nonpharmacological treatments (eg, physical therapy) for chronic musculoskeletal pain. During the study, we will systematically document participants’ receipt of usual care in all study arms, including medications, physical therapy, and clinic visits that can help pain (eg, pain specialist, psychologist, and acupuncture). A usual care arm allows us to estimate retention rates of not receiving any active treatment in the trial, informing the choice of a control condition for the planned adequately powered RCT to follow.

### Safety and Treatment Fidelity

Listening to music can at times bring up strong emotions, which can increase stress. The potential of this risk is minimal because of the supportive structure of the interventions and the overall study. The therapists are trained to help participants manage any strong emotions that emerge and, if needed, will refer participants for additional psychosocial support.

At each follow-up assessment (1 month, 3 months, and 4 months), a member of the research team will systematically check for participant intercurrent illness, adverse events, and serious adverse events. At all assessments, including baseline, a member of the research team will screen for suicidal ideation guided by a 3-step algorithm consisting of the following: (1) item 9 suicide question of the Patient Health Questionnaire-9 (PHQ-9), (2) the Columbia Suicide Severity Rating Scale Screener if item 9 of PHQ-9 is positive, and (3) Comprehensive Suicide Risk Evaluation for moderate to high risk of suicide based on the Columbia Suicide Severity Rating Scale Screener [43-45].

To ensure intervention fidelity and minimize experimental drift, we will use the following strategies: (1) standardized intervention protocols, study manuals, and training; (2) self-monitoring of intervention sessions recorded in field notes with debriefings across music therapists; (3) quality assurance checklists to track protocol deviations; and (4) therapist field notes documenting session duration and frequency (dose).

### Data Collection and Measures

#### Overview

After obtaining informed consent, the project coordinator will administer a baseline assessment to gather sociodemographic, medical, and psychological comorbidities and prior experience with music; review the patient’s treatment history emphasizing previous treatments tried for chronic musculoskeletal pain; and administer measures to assess pain, function, psychological status, and other relevant constructs.

The conceptual framework that informed the choice of study measures is illustrated in Figure 1. The *antecedents* are the patients’ pre-existing conditions that will serve as potential covariates. The music interventions are expected to improve outcomes both directly and indirectly through several theoretically derived *mediators*. During therapist-delivered sessions, more engagement is expected to improve mood, enhance therapeutic alliance, and increase self-efficacy, leading to greater independent practice and use of music [17]. Compared with music listening, the music imagery intervention draws upon a patient’s inner resources (creativity, courage, and serenity) to improve resilience and perceived benefit. The outcomes are based on a biopsychosocial model of pain and include several measures that encompass the biological, psychological, and social dimensions of pain. The mechanistic pathways depicted offer a theoretical framework for the intervention. Although the pilot is insufficiently powered to find significant mediation, pathway testing will be conducted to help inform the future larger RCT.

Assessments will be conducted by video interviews, unless phone or face-to-face interviews are preferred by the veteran. Assessments of similar lengths have been used by the researchers in several previous trials without overburdening the participants. Following each music imagery session, we will collect brief feedback on the content and delivery of the session. To assess immediate posttreatment effects, we will administer a pre-post visual analog scale to assess change in patient-identified symptoms (eg, pain and anxiety).

We will conduct qualitative interviews with up to 20 veterans following the completion of their last session and assessments with a minimum of 8 participants from each music intervention arm. These interviews will assess the participants’ perceptions of the interventions and study participation.

#### Aim 1 Outcomes

Feasibility metrics include recruitment, retention, completion in allotted time (eg, 12 weeks for the music imagery group), and completion of assessments. The goal is to recruit and enroll 60 veterans into the pilot trial and to complete a median of 80% of all study visits. Those who miss visits will be called upon to ask about barriers to attendance. Individuals who dropped out of the study will be asked what could have been done to improve their participation. music imagery sessions will occur weekly on a fixed schedule while allowing some flexibility in scheduling for the participant’s convenience. The proportion of music imagery participants who complete all sessions within the treatment period will be measured. On the basis of the previous trials, the expected completed outcome assessments rate is 85%.

Acceptability of randomization to study groups will be tracked. Treatment credibility will be assessed for each music intervention and veterans’ treatment satisfaction, experience of the interventions, benefits, and barriers or facilitators to study participation will be gathered through qualitative interviews at the end of the treatment period. Safety of the music interventions will be assessed by tracking exacerbations of pain, depression, anxiety, and any adverse events.

#### Aim 2 Outcomes

The selected measures for aim 2 are outlined in Table 1. The choice of measures is informed by the Initiative on Methods, Measurement, and Pain Assessment in Clinical Trials recommendations, our previous studies, the biopsychosocial model, Creative Forces Network’s conceptual framework for music therapy, and constructs hypothesized to be affected by the interventions [37].

**Table 1:** FAMILIA (Feasibility and Acceptability of Music Imagery and Listening Interventions for Analgesia) patient-centered outcomes: measures and schedule of administration.

Domain		Measure	Schedule							
			BL^a^		1 month		3 months		4 months	
**Antecedents**										
	Covariates	Demographics, military history, disability compensation, comorbidity, pain treatments	✓^b^							
**Potential mediators**										
	Therapeutic relationship	Working Alliance Inventory					✓			
	Inner resources	State-Trait Assessment of Resilience Scale	✓		✓		✓		✓	
	Self-efficacy	Arthritis Self-Efficacy Scale	✓				✓			
**Outcomes**										
	Pain intensity	Numeric Rating Scale	✓		✓		✓		✓	
	Pain interference	Brief Pain Inventory-interference	✓		✓		✓		✓	
	Pain beliefs	Brief Pain Catastrophizing Scale	✓				✓			
	Pain coping	Centrality of Pain Scale	✓				✓			
	Pain treatment response	Patient Global Impression of Change			✓		✓		✓	
	Sleep	PROMIS^c^ Sleep Scale	✓		✓		✓		✓	
	Depression	PHQ-9^d^ Depression	✓		✓		✓		✓	
	Anxiety	GAD-7^e^ Anxiety	✓		✓		✓		✓	
	Trauma	PC PTSD^f^ Screen	✓							
	Stress	Perceived Stress Scale	✓				✓			
	Generic HRQL^g^	EQ-5D	✓				✓			
	Social support	Brief Social Support Scale	✓							

^a^BL: baseline.

^b^Check marks indicate when the measure is performed.

^c^PROMIS: Patient-Reported Outcomes Measurement Information System.

^d^PHQ-9: Patient Health Questionnaire-9.

^e^GAD-7: 7-item Generalized Anxiety Disorder.

^f^PC PTSD: primary care posttraumatic stress disorder.

^g^HRQL: health-related quality of life.

#### Brief Description of Proposed Measures for FAMILIA Pilot Study

##### Potential Mediators

The following measures were chosen to explore potential mechanistic pathways for the intervention:

1. Working Alliance Inventory: the Working Alliance Inventory is a measure of the therapeutic alliance that assesses three key aspects of the therapeutic alliance: (1) agreement on the tasks of therapy, (2) agreement on the goals of therapy, and (3) development of an affective bond [46].
2. State-Trait Assessment of Resilience Scale: this new measure assesses resilience with a state and trait approach. Early studies suggest that the State-Trait Assessment of Resilience Scale is a useful measure to track and predict an individual’s resilience [47].
3. Arthritis Self-Efficacy Scale: this 8-item scale proved sensitive to change in our trial of low back pain. For each item, patients reported their degree of certainty on a scale ranging from 1 (very uncertain) to 10 (very certain) [48].

##### Outcomes

The following outcome measures encompass the biological, psychological, and social dimensions of pain:

1. Numeric Rating Scale: this pain intensity scale uses a 0 to 10 numeric rating scale that is anchored with “0”=no pain and “10”=the worst pain imaginable. The Numeric Rating Scale has been validated for specificity and use in chronic pain research [49].
2. Brief Pain Inventory (BPI): the BPI is a multidimensional pain measurement tool with demonstrated reliability in patients with arthritis and other pain conditions. The BPI rates the intensity of pain as well as the interference of pain with mood, physical activity, work, social activity, relationships with others, sleep, and enjoyment of life [50].
3. Brief Pain Catastrophizing Scale: this new brief version of the Pain Catastrophizing Scale has been derived and shows sound measurement properties and strong association with the full 13-item original scale [51].
4. Centrality of Pain Scale: the Centrality of Pain Scale is a 10-item self-report instrument. Responses are measured on a 5-point Likert scale ranging from “strongly disagree” to “strongly agree.” In its original validation study, the scale demonstrated high internal consistency (Cronbach α=.90) and good construct validity [52].
5. Patient Global Impression of Change: this is a 15-point, well-validated, global rating scale for evaluating changes in symptoms over time. This scale asks patients to make an initial global assessment of change in their symptoms as “worse,” “about the same,” or “better.” Those who respond that they are “worse” or “better” are asked to make an additional global rating [53].
6. The Patient-Reported Outcomes Measurement Information System Sleep Scale: the Patient-Reported Outcomes Measurement Information System Sleep Disturbance Scale assesses sleep quality and depth, along with difficulties and satisfaction with sleep. Scores are standardized to the general United States adult population on the T-scale with a mean of 50 and SD of 10. Higher scores indicate more symptoms being assessed. A clinically meaningful difference is considered to be ≥5 points [54].
7. PHQ-9 depression: several studies have validated the PHQ-9 as a diagnostic measure with excellent psychometric properties. Internal consistency has consistently been shown to be high (Cronbach α>.80), and test-retest assessment showed the PHQ-9 to be a responsive and reliable measure of depression treatment outcomes [43].
8. 7-item Generalized Anxiety Disorder-Anxiety: the 7-item Generalized Anxiety Disorder has demonstrated reliability (Cronbach α=.89) and validity (criterion, construct, and factorial) as a measure of anxiety in the general population and in primary care [55].
9. Primary Care PTSD Screen: the Primary Care PTSD Screen for the Diagnostic and Statistical Manual of Mental Disorders, Fifth Edition is used as a screening measure, and is a modified version of the PTSD module of the MINI-International Neuropsychiatric Interview to diagnose Diagnostic and Statistical Manual of Mental Disorders, Fifth Edition PTSD. It has demonstrated strong preliminary results for diagnostic accuracy [56].
10. Perceived Stress Scale: the 10-item Perceived Stress Scale is the most widely used psychological instrument for measuring the perception of stress and has been used in previous music trials [57].
11. EQ-5D-5L: the EQ-5D-5L questionnaire is an instrument for describing and valuing health states and has been used in hundreds of trials. It is a brief self-reported measure of generic health that consists of 5 dimensions (mobility, self-care, usual activities, pain or discomfort, and anxiety or depression), each with 5 levels of functioning [58].
12. Brief Social Support Scale: this reliable and valid short (6-item) scale assesses the emotional and practical dimensions of social support [59].

### Data Analysis

As an RCT, it is expected that baseline characteristics of the study participants will be balanced across the 3 treatment groups; however, possible group imbalances will be assessed. Outcomes (feasibility, acceptability, pain, and other patient-centered outcomes) will be reported as means with SDs for continuous variables, or medians (IQRs) for nonlinear data and frequencies with percentages for categorical variables. Changes in pain, associated patient-centered outcomes, and proximal and distal mediators will be explored from baseline to follow-up assessments (1, 3, and 4 months). Bivariate analyses will be performed first to determine if there are significant differences in the 3 treatment groups for the antecedent, mediator, and outcome variables, which will also help to determine if there are group imbalances as discussed above. These analyses will be performed using ANOVA and chi-square tests for continuous and categorical variables, respectively. No post hoc *P* value adjustments will be made, as this is a feasibility study. Data transformations will be used if necessary and Fisher exact test will be used instead of chi-square when expected cell counts are small, with data analyses being performed using SAS software (version 9.4; SAS Institute [60]). Path analyses will then be performed to examine the path coefficients for the entire conceptual study model (Figure 1). The path coefficients, model fit indices, and covariance structures, along with the results from the bivariate analyses, will be used to determine model adjustments and parameter estimates necessary to determine the power for a larger full study. Path analyses will be performed using Mplus (version 8.7; Muthén & Muthén [61]).

Using guidelines from the thematic analysis by Braun and Clarke [62], the qualitative analysis of veteran interviews and music imagery participant listening logs will occur in 2 broad phases: open coding and focused coding. Open coding facilitates development of a code list for further analysis. In this phase, selected transcripts are read to gain a general understanding of the data and variation across participants. Then, each line will be independently labeled with initial codes to describe the data. The research team will meet to discuss and compare initial codes. The team will then examine the codes, looking for emerging categories to sort and organize the data. As new transcripts are reviewed and coded, categories may be altered or codes are restructured within the categories. This process will occur iteratively until agreement on, and shared understanding of, emergent thematic categories is established. In phase 2, focused coding, the categories derived in the first phase will be applied to transcripts. A subset of transcripts will be coded by all analysts using the agreed-upon categories. Team members will meet in pairs to ensure coding consistency, with discrepancies resolved by consensus. Representative quotations will be presented for each category.

### Ethics Approval

FAMILIA was reviewed and approved by the Indiana University institutional review board and the VA research and development committee (IRB #12794).

## Results

### Participant Involvement in the Research Design

The FAMILIA research team consulted with the VA Health Services Research & Development Service Pain/Opioid Consortium of Research Veteran Engagement Panel facilitation team and outlined a plan for how the panel can inform this project. The panel group includes 12 veterans from across the country who serve as patient advisors to researchers to enhance patient-centered research. All panel members have personal experience with chronic pain or opioids. Veteran members are a diverse group of men and women across a wide age range and include students, retirees, and current workers with varied military, volunteer, and professional experiences. Panel members provided patient-centered feedback that helped inform the study recruitment materials, study design, and anticipated feasibility issues.

### Preliminary Data

Members of the research team conducted a single-arm study of 8 veterans with chronic pain to develop and pilot the telehealth MI intervention proposed for FAMILIA. Qualitative data were collected regarding the accessibility and feasibility of virtual delivery and the outcome measures of pain, depression, and anxiety. Findings from this small pilot study helped refine the music imagery intervention and its delivery for FAMILIA. For example, owing to some veterans’ lack of technology skills, a technology questionnaire was added to the eligibility screening.

### Potential Results and Interpretations

There are several potential results and interpretations from the trial. It is expected that both music interventions will prove acceptable to veterans and that feasibility benchmarks will be reached. However, it is possible that one or both music interventions will not be embraced by all participants or may not meet all specified benchmarks. The qualitative interviews will identify the range of participant perceptions and experiences of both interventions and provide insight into the reasons for unacceptability or feasibility concerns. Although it is hypothesized that both music interventions will be more effective than usual care on pain and associated outcomes, differences in outcome scores may be observed but are unlikely to reach stochastic significance (because FAMILIA is not powered to evaluate efficacy). This potential scenario is acceptable in the context of pilot clinical trials where the primary objective is to field-test logistical aspects of the future RCT and to incorporate these aspects into the study design.

### Trial Status

FAMILIA received institutional review board approval in October 2021 and approval from VA Research and Development in November 2021. Recruitment began in March 2022 and as of July 2022, a total of 16 participants were enrolled.

## Discussion

The primary purpose of FAMILIA is to examine the feasibility and acceptability of a multicomponent interactive intervention (music imagery) and a single-component, minimally interactive listening intervention (music listening). FAMILIA is not powered to evaluate efficacy, but we anticipate that the music interventions will have a greater effect on outcomes compared with usual care.

### Strengths and Limitations

FAMILIA not only addresses the limitations of prior research but also responds to important clinical and research initiatives to develop and test safe and effective nonpharmacological interventions for chronic pain. In clinical practice, analgesics are widely used for pain, but suboptimal pain relief is common as are the side effects. Adverse events related to analgesics, especially nonsteroidal anti-inflammatory drugs and opioids, are well known. Given the modest benefits of analgesics and their safety concerns, novel nonpharmacological options are needed to reduce clinician and patient reliance on analgesics and to reduce the enormous burden of chronic pain on patients.

We selected an active comparator (music listening) to inform whether the design of the subsequent RCT should be a comparative effectiveness trial. We also designed FAMILIA to include a usual care control rather than other comparator options (eg, wait-list control or attention control). A usual care control group is appropriate given the paucity of studies and limited efficacy data of music interventions for chronic pain, especially for virtually delivered interventions. The study sample was drawn from a single VA medical center and may not be representative of all patients with chronic musculoskeletal pain.

### Conclusions

The long-term objective of this research team is to develop and test music interventions that improve chronic pain management and can be implemented in various clinical settings. Findings from FAMILIA will inform refinement of interventions and trial design in advance of conducting a fully powered trial to identify putative mechanisms and test efficacy.

## Appendix

Multimedia Appendix 1 External review report from grant funders.

## Abbreviations

BPI: Brief Pain Inventory
FAMILIA: Feasibility and Acceptability of Music Imagery and Listening Interventions for Analgesia
ICD 9/10: International Classification of Diseases 9th and 10th editions
PHQ-9: Patient Health Questionnaire-9
PTSD: posttraumatic stress disorder
RCT: randomized controlled trial
VA: Veterans Affairs

## Data Availability

Owing to privacy and ethical considerations, final data sets underlying publications resulting from this research will not be shared outside the Department of Veterans Affairs, except as required under the Freedom of Information Act.
