# Supplementary material for: Feasibility and Acceptability of Music Imagery and Listening Interventions for Analgesia: Protocol for a Randomized Controlled Trial
Source: JMIR Res Protoc. 2022 Sep 22;11(9):e38788. doi: 10.2196/38788 (PMC9539652; doi:10.2196/38788)
Supplement: Multimedia Appendix 1 [file resprot_v11i9e38788_app1.pdf]

ART THERAPY & MUSIC THERAPY RESEARCH WITH MILITARY POPULATIONS
CLINICAL RESEARCH APPLICATION FEEDBACK

Bair and Story: Feasibility and Acceptability of Music Imagery, and Listening Interventions for Analgesia (FAMILIA)

Strengths

- Design of the 3-arm feasibility study is based on a biopsychosocial framework designed to evaluate the effectiveness of one-to-one sessions examining music and imagery compared to music listening protocol and usual pain management care. This design approach will help inform and isolate two music therapy interventions as to effectiveness in treating chronic pain and outcomes.
- The preliminary work with 10 Vets in the single arm study adds credence to the proposal.
- The weakness of one site is identified and addressed with possibly adding Denver.
- The proposed study aligns with Creative Forces' prioritized research question by examining the feasibility and acceptability of MI and ML in treating chronic pain among Veterans.
- The research design and methodology are scientifically justified and appropriate to address the research questions for the feasibility/pilot study and can lead toward a RCT or large-scale study.
- The feasibility, acceptability, and outcome measures seem appropriate and including both qualitative and quantitative measures is a strength. They also carefully considered participant burden when designing the study and estimate it will only require about 20 to 30 minutes to complete measures at each time point. They also will be compensating participants for their time.
- In preparation for this submission, the team also consulted with the VA HSR&D Pain/Opioid Consortium of Research (CORE) Veteran Engagement Panel (VEP) facilitation team and outlined a plan for how the VEP can inform this project. They will meet with the VEP multiple times during the proposed research. These consultations will embed a Veteran-centered lens throughout the design, implementation, and interpretation of our research.
- Excellent team! There is notably a well-rounded team between MT, PhD, and MD. Team members all have extensive history in this area of practice and research. I am especially impressed with Whitmire, and I have no doubt about this team.

Section I. Scientific Merit

Significance/Relevance to Military Populations

- Chronic pain is complex and is influenced by the interaction of multiple biopsychosocial process, which the investigators noted. The quality and severity of pain can vary considerably between and within individuals, including Veterans. Although focusing on chronic pain in general will increase the generalizability of the findings, it may be difficult to detect statistically significant changes in the proposed outcomes due to the between- and within-person variability in chronic pain experiences. This may be more of an issue for Aim 2 and the large-scale RCT.

Research Question/Hypothesis, Rationale, and Progression

- While this proposed research study does align with Creative Forces prioritized research question of "how and to what extent dos music therapy affect the discomfort and disability associated with chronic pain in service members and/or Veterans experiencing chronic pain, it focuses on chronic musculoskeletal pain which may be a more broad category of pain caused by injury to bone, muscles, nerves and/or connective tissue. Creative Forces research priority if focused primarily on chronic pain with patient populations of military/Veterans suffering from mild traumatic injury or PTSD. This study however has implications in this focus population but may provide broader overall perspective reaching broader Veteran populations beyond mTBI/PTSD symptoms.
- I have the same concern as the first section. Chronic pain is complex, which the investigators noted, and is influenced by the interaction of multiple biopsychosocial process. The quality and severity of pain can vary considerably between and within individuals. Although focusing on general chronic pain will increase the generalizability of the findings, it may be difficult to detect statistically significant changes in the proposed outcomes due to the between- and within-person variability in chronic pain experiences at baseline. This may be more of an issue for Aim 2 and the large-scale RCT.

Research Design and Methodology (including resources such as budget)

- Within the research design providing more information on what will entail on questions for qualitative data for interviews would strengthen the proposed study as it is ambiguous in the proposed study.
- Research Strategy does not discuss the outcomes tools for quantitative analysis within the proposed study. Adding in outcome measure/tools would strengthen to proposed study and would demonstrated the integration of the tools to support the aims of the project. The proposed study has intervals of 1, 3 and 4 months to measure outcomes and 4 months may be too short and should be extended for listening groups and music and imagery groups (six months may provide better statistical outcomes).
- There should be a prescription of time associated (Dose) of time for each type of intervention.
- How do participants listen to music? Type of listening devices should be defined as there could be variances with users if using different equipment (i.e. Bose quiet no canceling headphones verses basic ear buds from Walmart.) Outcomes may be different for participants if not using the same equipment/products.
- Should have more control on listening groups and imagery and music – longer time to evaluate outcomes and to be sure study does not have bias for (music and imagery).
- The three time points are important (vs fewer); however, the longest time point post-evaluation is at the 4-month mark. AHRQ looks at chronic pain outcomes of fewer than 6 months to be a short-term benefit. Four months is not an adequate time, as in the acute phase, the scientific community could be misled into benefit. Does the benefit go away completely? What is the extinction rate? There is not a proposal for further follow-up to look at this, or to consider if a shorter "refresher" therapeutic course would buoy benefit. This is a major shortcoming.
- It would be beneficial to ascertain the Veterans' relationship with music. Do they have formal music training? Do they regularly listen to music? If so, genre and time input? When they participate in the study, what if they embark on other self-care (massage, meditation), which may confound results?

- There is not a prescribed time (“dose”) for the ML arm. This is stated but is actually a major issue. This is essentially a dose/response curve, and the pharmacokinetics and pharmacodynamics of music must be understood. Simply keeping a journal is not adequate, the music should be dosed.
- Besides doing of music, there is concern of active vs passive listening. The MI arm will force active listening and participation. One can passively or actively listen to music, and without being prescriptive to active listening (undivided attention), it biases towards MI.
- The MI group has 8 weekly sessions for 45 minutes. While there is a paucity of literature on best practice, this “novel-ish” approach should have more time afforded. Other work in this space utilizes 60-90 minutes, and I fear that 45 minutes is too short of an exposure time. The 8 sessions, as divided, also seem to not encapsulate enough time. Notably Stage 1, sessions 1 through 4 are a learning phase for self-regulation. Thus, Stage 2, sessions 5-8 are essentially the intervention or therapeutic phase. I am not convinced that that 4 sessions of deepening one’s inner resources can impact lasting change (especially not beyond 4 months).
- I am confused about the budget for headphones, how many headphones? What type of headphones? Are they shared? The listening experience is drastically differenct based on this, and biases towards MI.
- The RCT document states 6 months for follow-up, everything else states 4 months, there is a discrepancy.
- With MI, and the amount of time and personnel investment, a cost-effective analysis would be an extremely useful sub-aim.
- The data analysis plan does not include inferential statistics. Although this is a pilot study with a small sample size, they should employ inferential statistics.
- They stated that “ Within strata, randomization with block sizes of 9 will be executed to ensure balance of key baseline characteristics (e.g., baseline pain intensity).” They did not state what baseline characteristics or the process they will use to determine the baseline characteristics to include. This is important information to include in the proposal.
- They did not state if they will be controlling for prior experience with music and music therapy.
- They could have provided more details about the qualitative data that will be collected.

Section II. Technical Merit – Feasibility/Pilot Study

Investigative Team and Project Management

- Slight, but I think for the rigor of this study, a PhD level biostats team member should be on the proposal.

Partnerships

- This research project has a newly established partnership with a creative forces therapist that has been developed within last year as this is a new site for NEA location. The music therapist however brings experience and advanced training in Guided Imagery and Music in delivery of music imagery interventions and has been practicing for past nine years. Based on the advanced training and focus within practice on GIM should support research’s team efforts for a successful research project.

- For RCTs there appears to be a need to develop more partnerships with other VA Medical Centers to expand the study's capacity for enterprise impact to study outcomes of chronic musculoskeletal pain to be able to generalize the results reaching larger patient sample size.
- The proposal opened a Pandora's Box by suggesting the second site, casting doubt on whether the study can be done at one site. I realize it is because of a potential homogeneous population – it would have been better to just plan for multisite.

Research Environment

- N/A

Data Management Plan

- They did not describe the archiving procedure.

Limitations and Alternative Approaches

- The study sample suggested for feasibility study is drawn from a single medical center and the research team acknowledged that the sample may not be representative of all patients with chronic musculoskeletal pain. To improve the plan for a RCT would be consideration of 2 site to include Denver VA. RCT research could be strengthened with larger N of participants and with large network of music therapist within VA medical center would strength study and generalizability of findings to add protocol with trained music therapist at minimum of 10 interested sites with approved thru R&D for more enterprise approach.
- While the approach is strong, I did not see several citations to defend the rationale.
- They did not provide enough detail about the upstream approach to addressing recruitment issues.
- Another limitation is the complex nature of chronic pain and how the quality and severity various considerably between- and within- individuals. This may limit the likelihood that they will find statistically significant changes over time.

Risk Management Plan

- N/A
